# The Alpha Angle

**DOI:** 10.2106/JBJS.23.01089

**Published:** 2024-09-16

**Authors:** Seper Ekhtiari, Olivia Fairhurst, Lily Mainwaring, Vikas Khanduja

**Affiliations:** 1 Division of Orthopaedic Surgery, University of Toronto, Toronto, Ontario, Canada; 2 School of Clinical Medicine, University of Cambridge, Cambridge, United Kingdom; 3 Young Adult Hip Service, Department of Trauma and Orthopaedics, Addenbrooke’s Hospital, Cambridge University Hospitals NHS Foundation Trust, Cambridge, United Kingdom

## Abstract

➢ The alpha angle was originally defined on magnetic resonance imaging (MRI) scans, using a plane, parallel to the axis of the femoral neck. However, much of the literature on the alpha angle has used radiographs or other imaging modalities to quantify the alpha angle.
➢ The measurement of the alpha angle can be unreliable, particularly on radiographs and ultrasound.
➢ If radiographs are used to measure the alpha angle, the circle of best-fit method should be used on multiple different views to capture various locations of the cam lesion, and “eyeballing” or estimating the alpha angle should be avoided.
➢ The cam lesion is a dynamic and 3-dimensional (3D) problem and is unlikely to be adequately defined or captured by a single angle.
➢ Modern technology, including readily available 3D imaging modalities, as well as intraoperative and dynamic imaging options, provides novel, and potentially more clinically relevant, ways to quantify the alpha angle.

Femoroacetabular impingement (FAI) is defined by pathological contact between the acetabulum and the femoral head-neck junction^1-3^. Articular cartilage damage, acetabular labral tears, and early-onset degenerative changes (in some patients) are potential consequences of this mechanical conflict. FAI can be described in terms of 2 morphologic abnormalities, pincer and cam-type, although patients may present with both (mixed-type FAI). The true prevalence of cam morphology is unclear, with estimates ranging from 5% to 75%^4^, and has variations across age, sex, ethnicity, the presence or absence of symptoms, and activity levels^5,6^. Cam-type FAI is characterized by asphericity of the femoral head due to an osseous prominence at the femoral head-neck junction^7^. In classic cam-type FAI, the prominent femoral head-neck junction impinges on the acetabular rim, causing damage to the peripheral cartilage and labrum, with movement of the hip joint^8^. However, FAI is a 3-dimensional (3D) deformity; hence, the exact movements causing the most impingement vary by case, according to the anatomical position of the lesion^9^.

FAI syndrome refers to the clinical scenario in which FAI leads to symptoms such as pain, restricted range of motion, and secondary changes to muscle length and tension^10^. This is particularly important when considering the impact of FAI syndrome on quality of life, given the higher prevalence of this condition in the athletic population^6^. Moreover, FAI syndrome carries a substantial economic burden on the individual and societal levels^11^. Evidence consistently demonstrates that early detection and surgical intervention improve outcomes in patients with FAI syndrome and may decelerate the progression to osteoarthritis^12,13^. Hip arthroscopy is among the most common surgical treatments to alleviate symptoms and restore function in patients with FAI syndrome^14,15^. Preoperative arthritis reduces the likelihood of benefit from hip arthroscopy in FAI syndrome, with lower postoperative scores and increased risk of conversion to arthroplasty^16^. Hence, early detection and appropriate management of FAI syndrome are critical. Importantly, surgical management should be reserved for carefully selected symptomatic patients, as morphology alone is not a sufficient indication for operative management^6^.

The current diagnosis and management of FAI syndrome in clinical practice rely on a combination of a positive clinical examination and characteristic imaging findings. The alpha angle, the most commonly reported radiographic measure of cam morphology^17^, was first defined in 2002 by Nötzli et al.^18^ using magnetic resonance imaging (MRI) (Fig. 1). Cam morphology is clinically important, as it can be a risk factor for the development of osteoarthritis (more so than isolated pincer morphology)^19^. In the original definition by Nötzli et al., the alpha angle was defined as the angle between the femoral neck axis and a line connecting the femoral head center with the point of beginning asphericity of the head-neck contour^18^. Specifically, a para-axial plane was used, parallel to the axis of the femoral neck and passing through the center of the femoral head; this radial view was defined in each individual from the initial coronal scout view^18^.

**Fig. 1:**
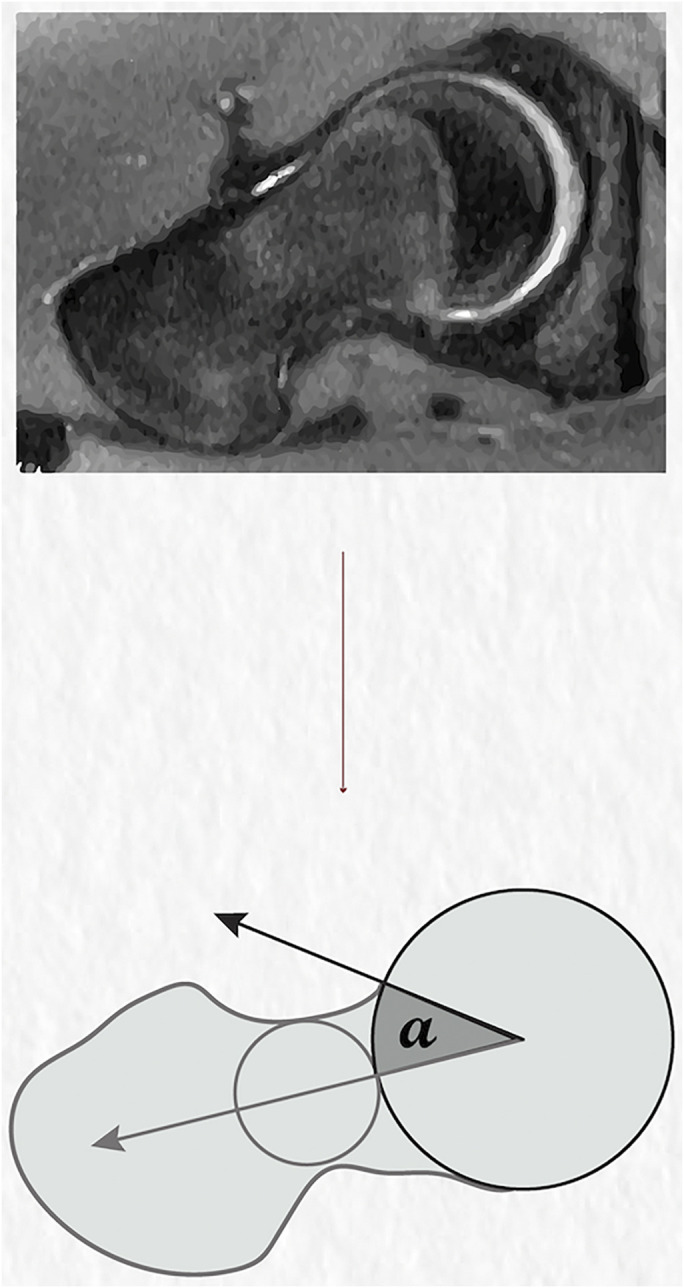
Top: MRI slice of the femoral head-neck junction. Bottom: Conceptual diagram demonstrating a sample alpha angle measurement using the circle of the best-fit method, with the alpha angle measured between the femoral neck axis and a line connecting the femoral head center with the point of beginning asphericity of the head-neck contour. The center of the femoral neck is determined as the center of a circle touching the cortices of the femoral neck on either side. Note that the measurement methodologies for both the femoral neck center and the alpha angle may vary slightly depending on imaging modality or planes; this diagram is simply meant to communicate the concept of the alpha angle. (Courtesy of Dr. Seyed Arad Mosalami Aghili.)

As hip preservation surgery becomes an increasingly common part of orthopaedic surgical practice and training^20^, it is important to revisit the clinical and academic utility of the alpha angle. Furthermore, with advancements in imaging technology and the increasing availability of 3D imaging modalities, it is important to consider the diagnostic, prognostic, and academic value of measuring a single angle to quantify a complex, 3D, and dynamic disease process.

## Which Imaging Modality Should We Be Using?

As with any diagnostic tool, accuracy and reliability are critical considerations; in other words, does the alpha angle accurately measure the head-neck junction cam deformity (i.e., accuracy), and can the angle measurement be reproduced by the same observers (i.e., intrarater reliability) and by different observers (i.e., interrater reliability)? For the alpha angle, accuracy and reliability depend on the imaging modality, the views or planes used, and the measurement techniques. Importantly, all accuracy and reliability metrics must involve comparison with a gold standard (ideally, the “truth,” but, in most cases, simply the modality originally used to define the measurement), and the selection of an inappropriate gold standard can also lead to misleading conclusions. The accuracy of the various imaging modalities is summarized in Table I, and the reliability metrics are summarized in Table II.

**TABLE I:** Studies Reporting the Sensitivity and Specificity of Different Imaging Modalities and Imaging Views or Planes*

Study	Study Type	Population	Pathological Cutoff	Comparator Imaging Modality (View)	Imaging Modality (View)	Sensitivity	Specificity
Robinson^49^ (2018)	Prospective	28 (11 patients had bilateral examination; 39 hips for analysis)	>55°	CT scan (axial oblique)	Ultrasound	91.3%	43.75%
Barton^23^ (2011)	Prospective	68 (43 FAI; 25 isolated labral tear controls)	50.5°	MRI scan (radial)	Radiograph (Dunn view)	91%	98%
					Radiograph (cross-table lateral)	74%	63%
					Radiograph (anteroposterior)	60%	81%
Nepple^37^ (2012)	Retrospective	41 (surgical patients with preoperative CT scans)	50°	CT scan (radial oblique)	Radiograph (Dunn 45° view)	80%	36.4%
					Radiograph (cross-table lateral)	40%	81.8%
					Radiograph (anteroposterior)	50%	81.8%
					Radiograph (frog-leg lateral)	46.7%	90.9%
Hellman^40^ (2015)	Retrospective	45 (FAI patients undergoing hip arthroscopy)	50.5°	CT scans (axial, coronal, and sagittal; 3D Slicer program)	Radiograph (false profile)	60%	89%
					Radiograph (90° Dunn lateral)	75%	89%
					Radiograph (anteroposterior)	25%	89%
					Radiographs (false profile, 90° Dunn lateral, and anteroposterior views combined)	86%	75%
Barrientos^59^ (2016)	Case-control	139 (38 cam or mixed-FAI; 101 controls)	≥57°	NA	CT scan (oblique axial)	92%	95%
Lohan^30^ (2009)	Retrospective	78 (39 intraoperative FAI; 39 asymptomatic controls)	55°	NA	MRI scan (oblique axial)	39.3%	70.1%

* NA = not applicable.

**TABLE II:** Intraobserver and Interobserver Reliability of the Alpha Angle Measurement, as Reported in the Literature, for Different Imaging Modalities and Imaging Planes*

Study	Study Type	Population Type	Imaging Modality (View)	Intraobserver Reliability	Interobserver Reliability
Mandema^47^ (2018)	Retrospective	32 (62 hips)	Ultrasound (supine, leg in neutral position)	Not reported	ICC, 0.74
Buck^48^ (2011)	Prospective	50 (patients with positive clinical exam for FAI)	Ultrasound (supine, leg in neutral position)	Not reported	ICC: 0.515 (anterior view); 0.509 (anterosuperior view)
Odri^33^ (2014)	Case-control	45 (26 FAI; 19 asymptomatic controls)	Radiograph (impingement position)	ICC, 0.88	ICC, 0.78
Cadet^35^ (2016)	Retrospective	8 (FAI, surgical management)	Radiograph (anteroposterior)	ICC, 0.34	ICC, 0.75
Agricola^41^ (2014)	Prospective	1,002 (CHECK cohort), 1,003 (Chingford study)	Radiograph (anteroposterior)	Kappa coefficient: 0.85 to 0.99 (CHECK cohort); 0.79 to 0.95 (Chingford study)	Kappa coefficient: 0.73 (CHECK cohort): 0.89 (Chingford study)
Konan^71^ (2010)	Retrospective	32 (FAI, surgical management)	Radiograph (frog-leg lateral)	ICC, 0.88	ICC, 0.83
Meyer^43^ (2006)	Retrospective	21 (dissected femora; 11 pathological and 10 normal)	Radiograph (cross-table 15° internal rotation)	Unpaired t test R, 0.97	Unpaired t test R, 0.97
			Radiographs (anteroposterior; 90° Dunn; Dunn 45° flexion; cross-table 15° internal rotation; cross-table neutral; external rotation)	Unpaired t test R for all views combined, 0.95	Unpaired t test R for all views combined, 0.88
Clohisy^42^ (2007)	Retrospective	80 (56 cam-FAI; 24 asymptomatic controls)	Radiograph (frog-leg lateral)	Kappa coefficient, 0.74	Kappa coefficient, 0.83
			Radiograph (anteroposterior)	Kappa coefficient, 0.60	Kappa coefficient, 0.85
			Radiograph (cross-table lateral)	Kappa coefficient, 0.73	Kappa coefficient, 0.56
Odri^33^ (2014)	Case-control	45 (26 FAI; 19 asymptomatic controls)	CT (medial para-axial)	ICC, 0.91	ICC, 0.86
Cadet^35^ (2016)	Retrospective	8 (FAI, surgical management)	CT scan (lateral)	ICC, 0.58	ICC, 0.09
Ng^34^ (2015)	Case-control	43 (12 symptomatic patients; 17 asymptomatic patients; 14 controls)	CT scan (oblique axial)	ICC, 0.948 to 0.957	ICC, 0.881
			CT scan (radial)	ICC, 0.929 to 0.972	ICC, 0.865
Nötzli^18^ (2002)	Case-control	74 (39 FAI; 35 asymptomatic controls)	MRI scan (oblique axial)	±3%	±7%
Ewertowski^28^ (2022)	Retrospective	19 (19 FAI)	MRI scan (radial)	Observer 1 (non-expert): ICC, 0.77; Observer 2 (expert): ICC, 0.93	ICC, 0.45
Golfam^29^ (2017)	Cross-sectional	200 (asymptomatic patients, 400 hips)	MRI scan (oblique axial and radial)	ICC, 0.84	ICC, 0.74

* ICC interpretation: <0.5 indicates poor reliability, 0.5 to 0.75 indicates moderate reliability, >0.75 to 0.9 indicates good reliability, and >0.9 indicates excellent reliability.

### MRI

#### Accuracy

**Fig. 2:**
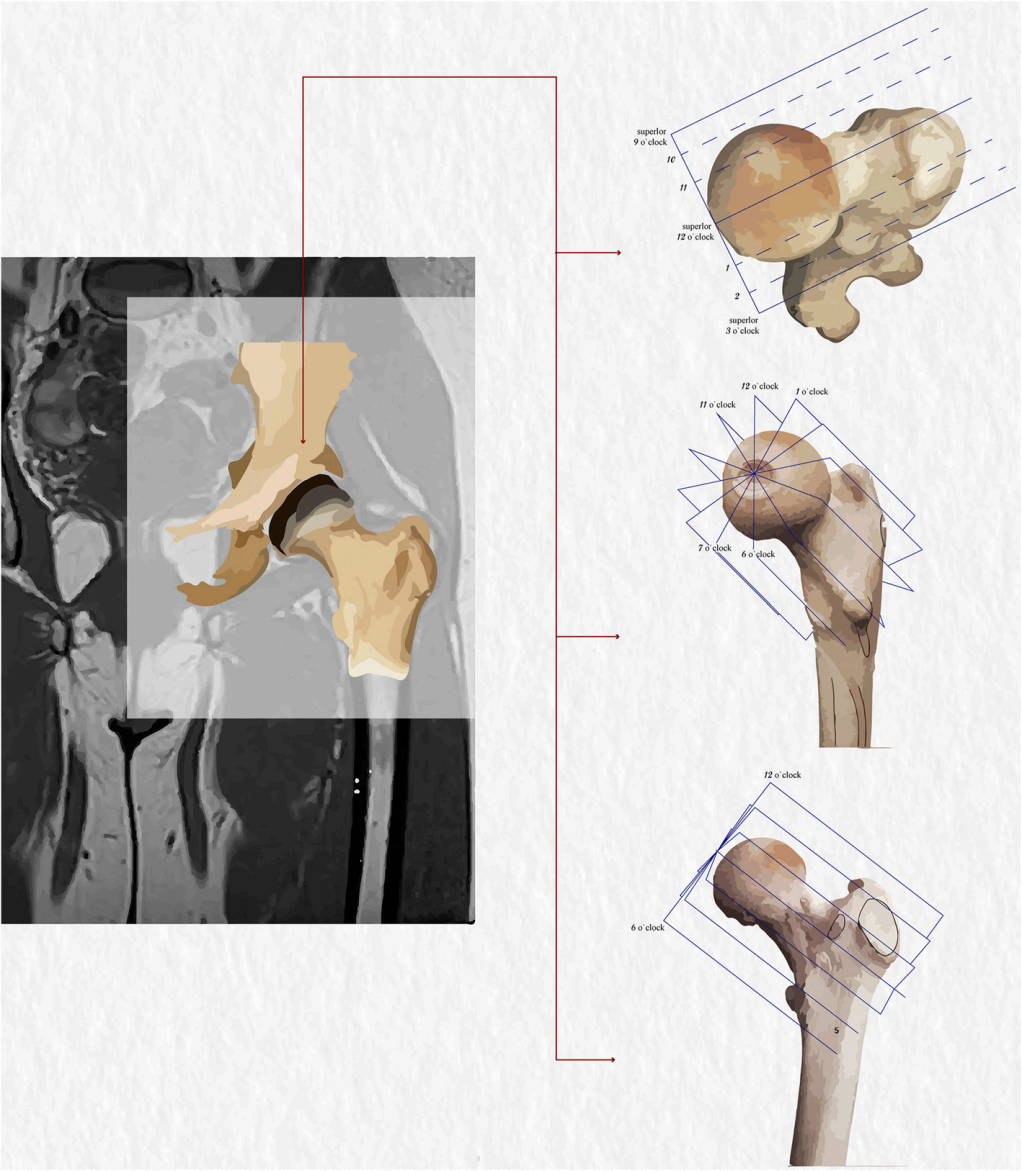
Radial MRI planes, which rotate around the axis of the femoral neck. The clock-face positions are also demonstrated. (Courtesy of Dr. Seyed Arad Mosalami Aghili.)

As the original imaging modality on which the alpha angle was originally described, MRI was the gold standard for measurement of the alpha angle^18,21^. The oblique plane used on MRI by Nötzli et al. (Fig. 2), the 12 o’clock plane, describes the relationship between the anterior femoral articular surface and femoral neck on MRI. This seminal article compared MRI scans of 39 patients who had groin pain, decreased internal rotation, and a positive impingement test, with 35 asymptomatic controls^18^. Another retrospective study of MRI scans from 40 patients with clinically positive FAI syndrome supported these findings, with 93% having abnormal alpha angles. Here, coronal and sagittal oblique planes were used^22^.

**Fig. 3:**
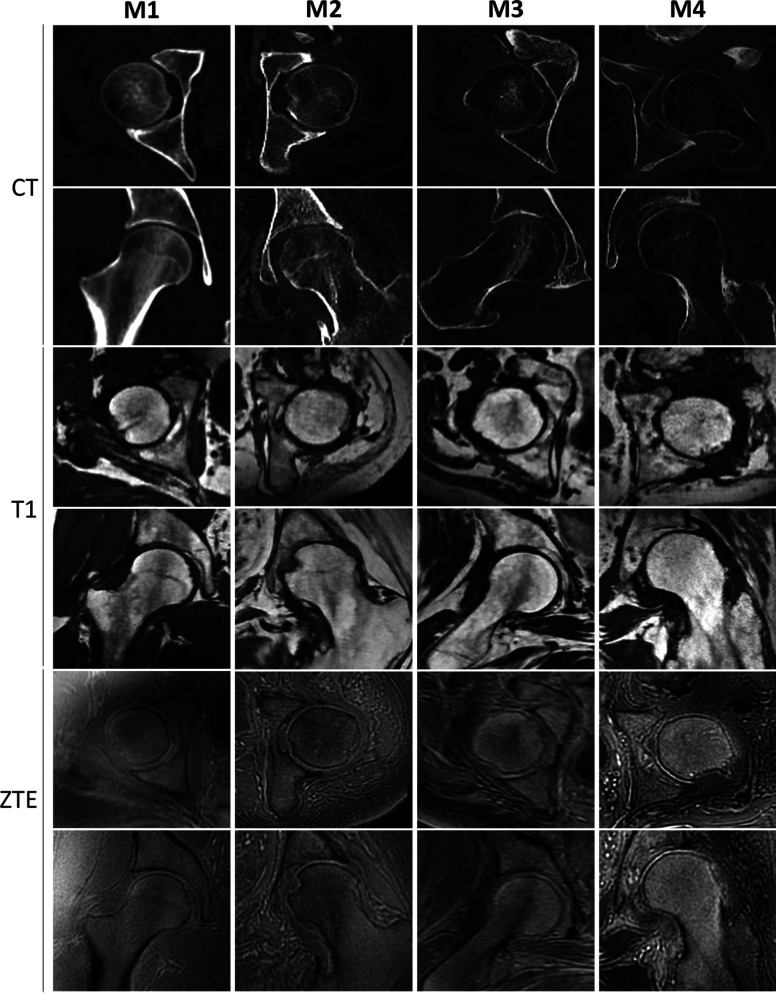
Osseous contour resolution as seen on CT scans, T1-weighted MRI scans, and ZTE MRI scans. (Reproduced from: Amar R, Thiry T, Salga M, Safa D, Chetrit A, Gatin L, Carlier RY. Comparison of magnetic resonance imaging and computed tomography for bone assessment of neurogenic heterotopic ossification of the hip: a preliminary study. J Orthop Surg Res. 2021 Dec 20;16[1]:725^27^, under a Creative Commons Attribution 4.0 International License.)

The MRI plane in which the alpha angle is measured must be carefully considered: higher alpha angle values are recorded in radial MRI scans when compared with oblique axial scans^23^. In radial MRI, the alpha angle is measured in multiple planes parallel to the femoral neck (Fig. 2). Indeed, the radial plane reportedly has improved sensitivity for the detection of cam deformities^24,25^, consistent with cam deformities having an increased prevalence at the anterosuperior femoral neck. Hence, MRI, using a radial plane, should be the gold-standard investigation for quantitative alpha angle measurement in FAI syndrome; however, radial-plane MRI scans are not routinely available or utilized, and not all radiologists are familiar with interpreting these images. Thus, clinical diagnosis of FAI syndrome requires a combination of history, clinical examination, and all available imaging modalities including radiographs. Cam lesions usually occur between the 12 and 3 o’clock positions on the radial MRI plane, with the peak alpha angle values most commonly presenting at the 2 o’clock position^25^. New zero echo time (ZTE) MRI scans have enabled bone morphology contrast comparable with that of computed tomographic (CT) scans, in addition to the direct visualization of cortical bone achieved by MRI (Fig. 3)^26,27^.

#### Reliability

Nötzli et al. reported an intraobserver variability of ±3% and interobserver variability of ±7% on MRI scans^18^. Similarly, Ewertowski et al. calculated good-to-excellent intraobserver reliability^28^, with an intraclass correlation coefficient (ICC) of 0.77 for the non-expert observers and 0.93 for the expert observers. The difference in observer expertise could explain the poor-to-moderate interobserver reliability calculated (ICC, 0.45). In an MRI study of 200 asymptomatic patients (400 hips), good intraobserver (ICC, 0.84) and interobserver (ICC, 0.74) values were demonstrated^29^. Conversely, in a retrospective analysis of MRI hip scans, Lohan et al. reported poorer intraobserver variability of up to 30% between the first and second alpha angle measurements for each subject^30^. They hypothesized that the preselection of patients with a positive impingement test and anterior hip pain in the study by Nötzli et al.^18^ could account for the discrepancy in their results^30^.

### CT Scans

#### Accuracy

CT imaging provides superior fine osseous detail when compared with MRI scans, and, importantly, it is fast and accessible and has a comparatively lower cost^31^. Smith et al.^32^ prospectively compared alpha angle measurements on CT and MRI scans, using the same planes as Nötzli et al.^18^, in patients with FAI syndrome, and found no significant difference in the alpha angle calculated on these 2 imaging modalities.

#### Reliability

Similar to MRI scans, CT scans show good reliability for calculating alpha angle values. A case-control study of 45 patients calculated an ICC of 0.91 for intraobserver reliability and 0.86 for interobserver reliability^33^. Near-perfect intraobserver reliabilities were calculated in both oblique axial CT planes (ICC, 0.948 to 0.957) and radial CT planes (ICC, 0.929 to 0.972) in a case-control study including 43 participants^34^. Moreover, excellent interobserver reliabilities were also calculated (ICC, 0.881 for oblique axial planes and 0.865 for radial planes). Conversely, Cadet et al. reported moderate intraobserver reliability (ICC, 0.58) and very poor interobserver reliability (ICC, 0.09) on CT scans^35^.

### Radiographs

#### Accuracy

Radiographs are inexpensive and easily accessible; thus, they are the most commonly reported means of evaluating and calculating the alpha angle^36^. However, different radiographic views result in alpha angle measurements taken at different locations on the head-neck junction. For example, an alpha angle calculated on an anteroposterior view quantifies the lateral head-neck junction, whereas the anterolateral head-neck junction is quantified by taking an alpha angle on a frog-leg lateral view or a Dunn view (which can be performed at 45° or 90°)^36^. Overall, both the 45° and 90° Dunn views have been reported to be more sensitive than cross-table lateral, anteroposterior, and frog-leg lateral views for identifying the presence of a cam lesion^23,37^ (Table I). However, the 45° Dunn view is prone to missing anterior cam lesions, for which the frog-leg view may be better suited^38^. The 45° Dunn view results in a significantly larger alpha angle measurement compared with the 90° Dunn view^32^. Clohisy et al. provided an excellent overview, including figures, demonstrating the correct radiographic techniques for various radiographic views^39^.

Odri et al. developed a new hip view, the “Profile view in Impingement Position” (PIP)^33^. PIP combines a lateral view with a false profile, resulting in a 65° angle between the femur and the pubic bone (adduction and flexion). The alpha angles were calculated for 26 patients with FAI syndrome and 19 controls in the PIP position on radiographs and compared with CT images: no significant difference was reported between radiographic and CT measurements^33^. Hellman et al. retrospectively reviewed preoperative radial oblique CT scans and radiographs of 45 patients undergoing hip arthroscopy^40^. Alpha angles were measured on radiographs using anteroposterior, 90° Dunn lateral, and false profile views and were compared with radial oblique CT scans, which were considered the “true” alpha angle. The mean alpha angle value of these 3 views was 86% sensitive for detecting cam-type deformities and, therefore, a better screening tool than a single radiographic view. Nepple et al. reported a sensitivity of 90% for the detection of abnormal alpha angles when taking the median of the anteroposterior, Dunn, and frog-leg lateral views^37^.

#### Reliability

Numerous studies examining alpha angle calculations on different radiographic views have reported good-to-excellent interobserver and intraobserver reliability^33,35,41-43^ (Table II). Agricola et al. combined 2 large prospective studies, examining nearly 3,000 hips in >2,000 individuals^41^. They reported a kappa value of up to 0.99 for intraobserver reliability and of 0.89 for interobserver reliability on anteroposterior pelvic radiographs.

Clohisy et al. reported a poorer intraobserver reliability for an anteroposterior view (ICC, 0.60) when compared with a frog-leg lateral view (ICC, 0.74) and a cross-table lateral view (ICC, 0.73)^42^. Meyer et al. reported that 45° and 90° Dunn views outperformed anteroposterior and cross-table lateral views in terms of reliability^43^. Odri et al. compared alpha angles calculated on radiographs and CT scans and reported a correlation coefficient of 0.73 and 0.8 for the 2 observers^33^. They also demonstrated good intraobserver and interobserver reliabilities for alpha angles calculated on radiographs in the PIP.

### Ultrasound

#### Accuracy

Ultrasound is a radiation-free means of imaging that is highly operator-dependent, and ultrasonography is not a routine part of orthopaedic training^44^. Furthermore, patient body habitus can impact the quality of images obtained by ultrasound, particularly when there are high levels of adiposity around the hip and pelvis^45^. Nevertheless, some of the comparisons with MRI have shown ultrasound to have promise for evaluating cam morphology. Lerch et al. compared alpha angles calculated on oblique axial MRI with those calculated on ultrasound examination in the ventral longitudinal section at 20° external rotation, neutral, and 20° internal rotation^46^. No significant differences were detected between the alpha angle calculated on MRI and ultrasound in neutral or 20° internal rotation. Mandema et al. found ultrasound to be a specific and sensitive means of assessing cam morphology^47^. However, this study compared ultrasound alpha angle measurements with those on anteroposterior radiographs, which have limited sensitivity and specificity themselves.

Buck et al. compared ultrasound with MRI and found that ultrasound performed poorly in alpha angle measurements. They hypothesized that this was due to the incorrect assumption that the femoral neck axis is parallel to a line drawn from the insertion of the joint capsule at the femoral neck to the center of the femoral head^48^. Moreover, misalignment of the ultrasound transducer could have led to further osseous contour distortion and measurement errors. Robinson et al. reported mixed results when comparing alpha angles calculated on ultrasound and CT, with ultrasound having a sensitivity of 91.3% and a specificity of 43.8%^49^.

#### Reliability

Buck et al. analyzed ultrasound images of the anterior and anterosuperior contours of the femoral neck obtained from 50 patients^48^ and reported moderate interobserver reliability (ICC, 0.509 to 0.515). In contrast, Mandema et al. reported good interobserver reliability, with an ICC of 0.74^47^. Those authors suggested that the lower interobserver reliability in the study by Buck et al. could be explained by their use of a curved probe, rather than a linear probe, resulting in less accurate imaging of the osseous contour.

## Safety

As imaging modalities that do not involve ionizing radiation, MRI and ultrasound have the best safety profiles of the imaging modalities discussed above^50^. Low levels of radiation are often a purported advantage of radiographs, particularly in comparison with CT. This is certainly a consideration in young adult patients undergoing hip preservation surgery, who have a mean age of 35 years^51^. It is certainly true that a single radiograph delivers far less radiation than a pelvic CT scan; a single anteroposterior pelvic radiograph involves 6% of the effective dose of radiation as a pelvic CT scan^52^. However, a single anteroposterior pelvic radiograph is not a sufficient workup for a young adult patient undergoing hip surgery. The Lisbon agreement on FAI syndrome has recommended an anteroposterior pelvic radiograph and a 45° Dunn view for the initial evaluation. The mean number of preoperative radiographs obtained for a patient undergoing hip arthroscopy is 5^53^, which cumulatively contain 36% of the dose of a pelvic CT scan^52^. Importantly, however, a series of 5 hip and pelvic radiographs actually has an effective dose that is 3.6 times higher than that of a low-dose CT protocol, which can provide sufficiently high-quality images for hip preservation surgeons^52^. Although, in many cases, radiographs and CT are performed sequentially and may both be necessary in the end, it is important that providers understand the relative effective radiation doses when deciding between imaging modalities. There is also substantial variation depending on institutional protocols and newer technologies that can automatically reduce the radiation doses delivered.

## How Should We Be Measuring the Alpha Angle?

**Fig. 4:**
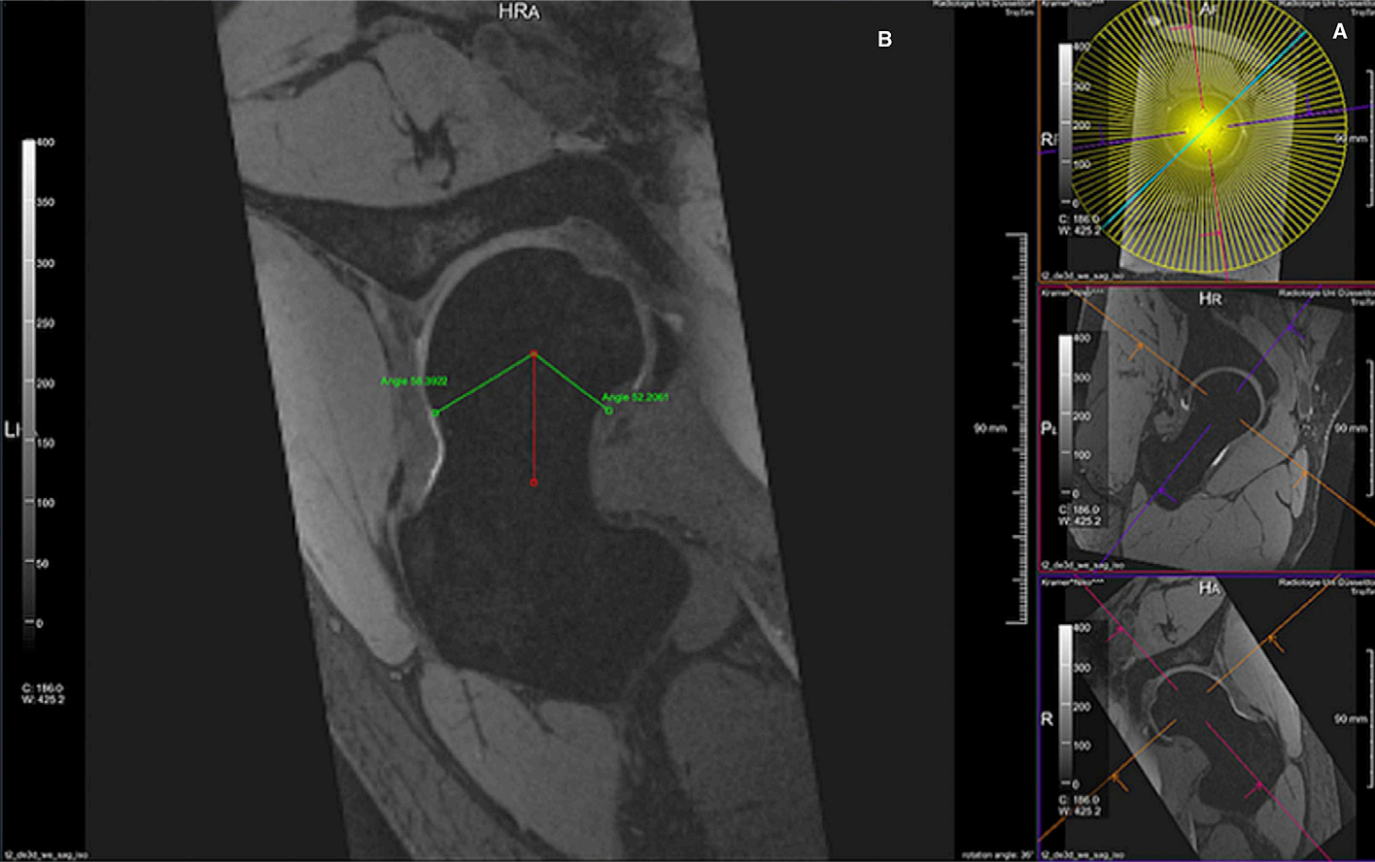
Alpha angle measurement using automated computer-based software (MR Chondral Health 2.1; Siemens Healthcare). **Fig. 4-A** There were 120 radial planes that could be used for alpha angle measurement. **Fig. 4-B** Alpha angle measurement performed by the software. (Reproduced, with labeling added, from: Ewertowski NP, Schleich C, Abrar DB, Hosalkar HS, Bittersohl B. Automated measurement of alpha angle on 3D-magnetic resonance imaging in femoroacetabular impingement hips: a pilot study. J Orthop Surg Res. 2022 Jul 30;17[1]:370, under a Creative Commons Attribution 4.0 International License.)

The method of measurement has important implications for the precision of alpha angle measurements. Multiple studies have found that “eyeballing” or guessing the alpha angle value is poor at distinguishing between a normal and an abnormal alpha angle^23,54^. Agricola et al. compared 2 different semiautomated techniques of measuring the alpha angle on radiographs from patients in 2 large cohorts^41^. The alpha angle in the CHECK (Cohort Hip and Cohort Knee) cohort was measured using 8 points to define the circle of best fit, followed by angle determination using statistical shape modeling (SSM) software. In the Chingford cohort, the circle of best fit was defined using 3 points, followed by use of validated MATLAB (The MathWorks)-based software. The authors found slightly better intraobserver reliability with the 8-point SSM compared with the 3-point MATLAB technique, but slightly higher interobserver reliability with the 3-point method, although a direct statistical comparison was not made^41^. Ewertowski et al. used automated software that targets cartilage segmentation on MRI scans to calculate the alpha angle (Fig. 4)^28^. This study suggested that an automated alpha angle calculation process was more reliable and time-efficient, which may be clinically useful^28^.

Automated alpha angle calculations can also be performed intraoperatively, to assist the surgeon in achieving optimal cam resection^55^. New technologies also allow dynamic, 3D CT analysis of femoral head morphology. Dynamic assessment can help to incorporate the entirety of the patient’s anatomy, including acetabular morphology, to determine if and where the cam lesion is problematic, which can be useful for both patient counseling and intraoperative decision-making. This software for measuring cam morphology showed sensitivity of 90% and specificity of 43%, although, again, the comparison was made to radiographs as a gold standard^56^. Intraoperative navigation is also a potentially useful tool for cam resection, resulting in significantly greater reductions in the alpha angle compared with conventional cam resection based on a randomized controlled trial; however, navigation is also associated with significantly longer positioning time and radiation exposure^57^.

**Fig. 5:**
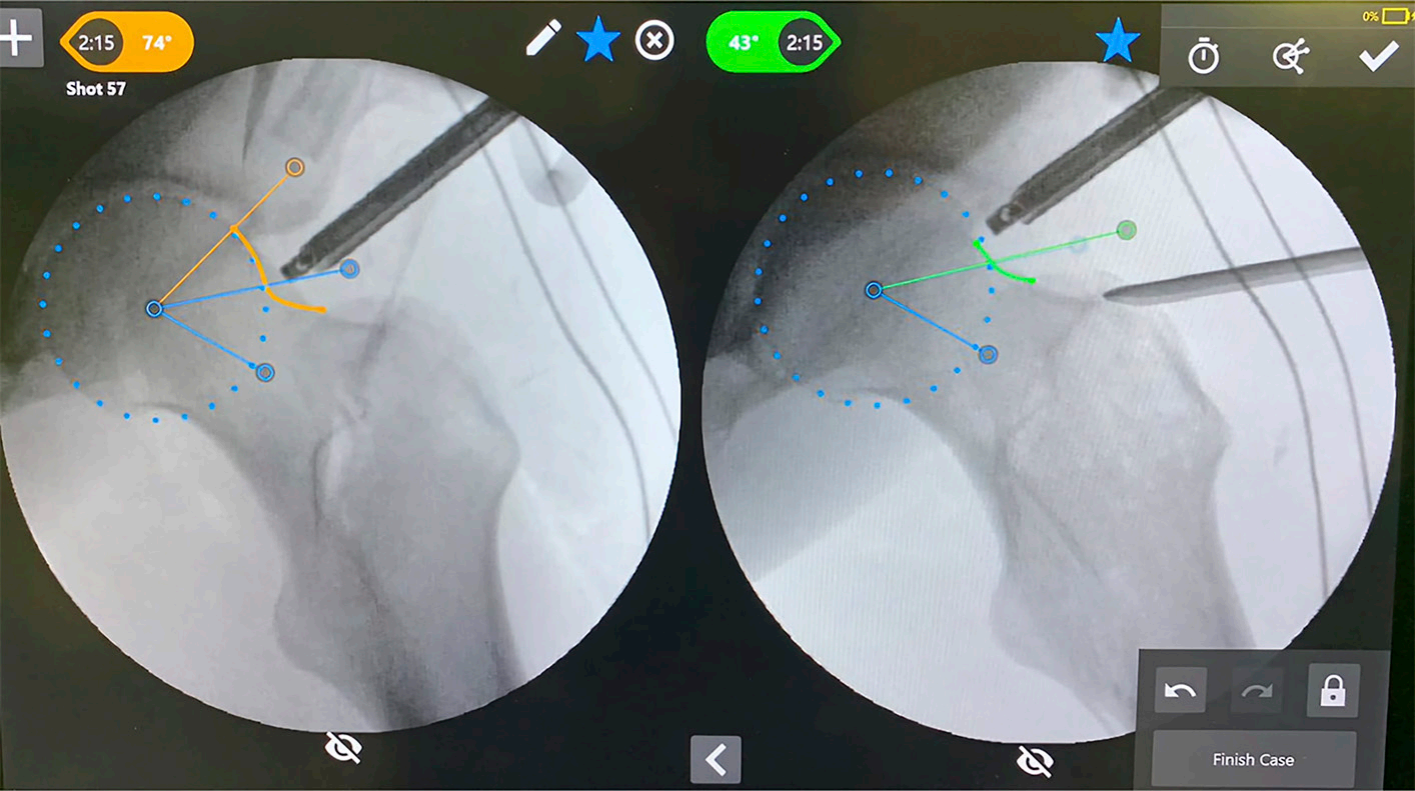
Intraoperative alpha angle measurement using the HipCheck system (Stryker).

Plastow et al. proposed a simple, 4-category classification system of cam lesions based on 3D CT scans of the head-neck junction. The 4 categories include anterolateral head-neck junction, anterolateral neck, anterior neck, and anterior head-neck junction (Fig. 5)^58^. Two senior surgeons in the study had perfect agreement on classification of the cam lesions (n = 91), and, when they were compared with a junior surgeon, there was 91% agreement. Intraobserver reliability ranged from 0.87 to 0.91. Thus, this classification system represents a potentially useful and reliable way to determine the type of cam lesion on 3D CT scans, in a clinically relevant manner^58^.

## What Is Normal?

An alpha angle of 55° is the commonly quoted diagnostic cutoff, based on the landmark article by Nötzli et al.^18^. The mean alpha angle in Nötzli’s FAI syndrome group was 74°. This is notable considering reports that surgical restoration of the alpha angle of <55° leads to improved outcomes in cam-type FAI syndrome^59^. However, subsequent studies have quoted different cutoff values for a pathological alpha angle, confusing both diagnostic criteria and the ability to compare studies^24,36,41,59-61^. Contributing to this are the conflation of FAI and FAI syndrome and differences between normative anatomic cutoffs for alpha angle and clinically important cutoffs for FAI syndrome.

In their retrospective study comparing 38 patients undergoing a surgical procedure for FAI syndrome with 101 controls, Barrientos et al. suggested a cutoff of ≥57°, arguing that this maximized sensitivity (92%) and specificity (95%)^59^. A more recent systematic review suggested a cutoff of ≥60° for defining a cam morphology, although the included studies were of generally low quality^36^. Sutter et al. found that increasing the diagnostic threshold from 55° to 66° decreased the number of false-positives in an MRI study on asymptomatic volunteers^24^. A study using an anatomic method (i.e., defining the femoral neck using multiple points, rather than 1 point as in the original method) found that this anatomic method resulted in a wider range of alpha angles, with a normal reference range of 30° to 66° in the general population^61^. “Normal” alpha angles ranging from 52° to 62° have been reported, with some studies finding higher mean values in men^60-62^. In a study of 2,005 hips, Agricola et al. reported a bimodal distribution of the alpha angle, and they suggested the implementation of 2 thresholds: 60° for the presence of a cam deformity and 78° for a pathological cam deformity, with the pathological cutoff based on the development of end-stage osteoarthritis on follow-up^41^. More recently, there has also been some consideration of the interplay between cam lesion location and femoral version^63^, as well as between cam lesion location and acetabular morphology (e.g., smaller alpha angles may still be pathologic in the context of acetabular retroversion)^64^.

## What Is the Utility of the Alpha Angle?

Alpha angle measurements from skeletal samples since the fourth century b.c. suggest an exponential growth in the prevalence of FAI, attributed to increases in height, weight, and body mass index^65,66^. One proposed etiology of cam lesions is developmental, being related to high levels of physical activity during skeletal development^67^. The most common reason for revision hip arthroscopy is residual FAI, specifically residual cam-type FAI^68,69^.

Lohan et al. found that using the alpha angle in a retrospective, blinded manner to evaluate patients with known treatment pathways offered no value in predicting the presence of cam lesions that would require surgical treatment^30^. They proposed 2 other measurements, 1 of which (the anterior femoral distance) did perform better than the alpha angle, although none of the measurements performed well enough to be considered useful in routine clinical practice^30^. This is likely because cam lesions are 3D deformities, and no single angle, or even combination of angles on planar imaging, can adequately capture the deformity.

In the practical, clinical setting, particularly when it comes to diagnosis and decision-making, the alpha angle value itself is likely less important than the presence or absence of a pathologic cam deformity. In this context, the presence or absence of a cam lesion, and the accurate determination of whether such a lesion is a source of the patient’s symptoms, is the most important question to be answered. From an academic perspective, the wide variability in accuracy, reliability, and techniques used to measure the alpha angle limits its interpretability and our ability to compare results across different studies.

It is clear that if the alpha angle is to be measured, it should ideally be measured on 3D imaging or, at the very least, on multiple different radiographic views, with an understanding of which anatomic locations are best visualized on each view. Measurement methods should use a circle of best-fit method rather than a simple angle, and (semi)automated measurements hold the promise of improved accuracy and reliability in a time-efficient manner. Table III summarizes our recommendations with corresponding grades of recommendation^70^.

**TABLE III:** Grades of Recommendation

Recommendation	Grade of Recommendation*
An MRI scan using a plane parallel to the femoral neck axis was the gold standard for the alpha angle as it was originally described and is the ideal comparator for quantitative measurement of the alpha angle^18,28-30^.	B
CT scans demonstrate similar accuracy and reliability to MRI scans in measuring the alpha angle^33-35,59^.	B
Radiographs have variably reported accuracy and reliability in measuring the alpha angle, but this improves when multiple views are used^23,33,35,37,40-43^.	C
Ultrasound has poor accuracy and low reliability for quantifying the alpha angle^47-49^.	C
When measuring the alpha angle, the circle of best fit using multiple points outperforms estimation or single-point measurement^41^.	B

* According to Wright^70^, grade A indicates good evidence (Level-I studies with consistent findings) for or against recommending intervention; grade B, fair evidence (Level-II or III studies with consistent findings) for or against recommending intervention; grade C, poor-quality evidence (Level-IV or V studies with consistent findings) for or against recommending intervention; and grade I, insufficient or conflicting evidence not allowing a recommendation for or against intervention.

Overall, the utility of the alpha angle as a single measurement in today’s hip preservation practice is unclear. A single angle is likely inadequate to capture a complex 3D and dynamic pathologic process, and the methods for measuring this angle are rife with imprecision and variable reliability. That being said, the alpha angle is still the most commonly used and reported method for quantifying cam deformity^17^. Also, it is important to correlate preoperative localization of the maximal alpha angle with direct intraoperative visualization of the lesion, as this can help to prevent under-resection. Although the alpha angle may continue to play a role in the diagnosis and management of FAI, the path forward will likely include much more 3D standardization, automation, and dynamic assessment of these measurements.
